# Opportunities for the Implementation of a Digital Mental Health Assessment Tool in the United Kingdom: Exploratory Survey Study

**DOI:** 10.2196/43271

**Published:** 2023-08-07

**Authors:** Benedetta Spadaro, Nayra A Martin-Key, Erin Funnell, Jiří Benáček, Sabine Bahn

**Affiliations:** 1 Cambridge Centre for Neuropsychiatric Research Department of Chemical Engineering University of Cambridge Cambridge United Kingdom; 2 Psyomics Ltd Cambridge United Kingdom

**Keywords:** assessment, digital mental health, development, implementation, mental health, provision, support, mobile phone

## Abstract

**Background:**

Every year, one-fourth of the people in the United Kingdom experience diagnosable mental health concerns, yet only a proportion receive a timely diagnosis and treatment. With novel developments in digital technologies, the potential to increase access to mental health assessments and triage is promising.

**Objective:**

This study aimed to investigate the current state of mental health provision in the United Kingdom and understand the utility of, and interest in, digital mental health technologies.

**Methods:**

A web-based survey was generated using Qualtrics XM. Participants were recruited via social media. Data were explored using descriptive statistics.

**Results:**

The majority of the respondents (555/618, 89.8%) had discussed their mental health with a general practitioner. More than three-fourths (503/618, 81.4%) of the respondents had been diagnosed with a mental health disorder, with the most common diagnoses being depression and generalized anxiety disorder. Diagnostic waiting times from first contact with a health care professional varied by diagnosis. Neurodevelopmental disorders (30/56, 54%), bipolar disorder (25/52, 48%), and personality disorders (48/101, 47.5%) had the longest waiting times, with almost half (103/209, 49.3%) of these diagnoses taking >6 months. Participants stated that waiting times resulted in symptoms worsening (262/353, 74.2%), lower quality of life (166/353, 47%), and the necessity to seek emergency care (109/353, 30.9%). Of the 618 participants, 386 (62.5%) stated that they felt that their mental health symptoms were not always taken seriously by their health care provider and 297 (48.1%) were not given any psychoeducational information. The majority of the respondents (416/595, 77.5%) did not have the chance to discuss mental health support and treatment options. Critically, 16.1% (96/595) did not find any treatment or support provided at all helpful, with 63% (48/76) having discontinued treatment with no effective alternatives. Furthermore, 88.3% (545/617) of the respondents) had sought help on the web regarding mental health symptoms, and 44.4% (272/612) had used a web application or smartphone app for their mental health. Psychoeducation (364/596, 61.1%), referral to a health care professional (332/596, 55.7%), and symptom monitoring (314/596, 52.7%) were the most desired app features. Only 6.8% (40/590) of the participants said that they would not be interested in using a mental health assessment app. Respondents were the most interested to receive an overall severity score of their mental health symptoms (441/546, 80.8%) and an indication of whether they should seek mental health support (454/546, 83.2%).

**Conclusions:**

Key gaps in current UK mental health care provision are highlighted. Assessment and treatment waiting times together with a lack of information regarding symptoms and treatment options translated into poor care experiences. The participants’ responses provide proof-of-concept support for the development of a digital mental health assessment app and valuable recommendations regarding desirable app features.

## Introduction

### Background

Mental health concerns in the United Kingdom are prevalent and growing [1]. More recently, the COVID-19 pandemic has led to further increases in the incidence of adverse mental health symptoms, substance use, and suicidal ideation in the United Kingdom and worldwide [2,3]. Treatment options exist, and they are often effective for most mental health conditions; however, underdiagnosis and misdiagnosis are common, resulting in inappropriate and ineffective treatment. In the United Kingdom, only 1 in 8 adults receives mental health treatment [4], and it is estimated that at least 75% of the people with poor mental health do not get access to the right treatment [5]. Readily available opportunities for mental health assessments or screening may be useful to identify concerns earlier, allowing for patient stratification and, in turn, personalized treatment plans and strategies. Data show that early identification and home treatment for mental health problems can decrease hospital admissions, reducing high-cost intensive interventions [6].

To this end, web applications and smartphone apps may offer an innovative and cost-effective way to improve mental health care detection and diagnosis. Indeed, mental health assessment apps have the potential to support individuals by screening for mental health symptoms and supporting self-management [7] while aiding primary care professionals and less experienced mental health workers in clinical decision-making. In fact, digital technologies can help alleviate the load on the health care system by providing individuals with subthreshold or mild mental health symptoms with self-help tips and psychoeducation, thereby reserving the limited and specialized services for patients with more severe and complex symptoms.

Interestingly, it has been shown that patients are more inclined to report severe mental health symptoms on technology platforms than to a health care professional (HCP) [8], and patients value the empowerment and autonomy that can be obtained via the use of a digital platform [9]. These technologies also have the potential to aid in engaging typically hard-to-reach patient populations by reducing stigma and facilitating help-seeking behaviors [10]. Importantly, the acceptability and efficacy of digital platforms for improving the reach, quality, and impact of mental health care have been previously demonstrated [11], with research on digital screening and monitoring technologies showing feasibility across a range of mental health conditions [12]. Despite this, evidence also suggests low user engagement [13] and a high dropout rate for mental health apps [14,15], particularly in real-world settings [16]. In this regard, further insights into the promise of digital technologies for mental health screening and assessment are required, particularly in regard to investigating existing unmet mental health care needs and establishing potential users’ views on using a mental health app.

### Objectives

To this end, we set to investigate the potential value proposition of a mental health assessment app. To do this, we conducted a study that was divided into two parts: (1) a web-based anonymous survey and (2) a series of web-based semistructured interviews. This paper presents the results from part 1 of the study. The aim of this study was to understand the current state of mental health care provision in the United Kingdom and to explore experiences with digital technologies for mental health. To this end, the first objective was to explore experiences with mental health care provision offered via health care services and the workplace over the last 5 years. The second and third objectives were to explore the use of, and interest in, digital technologies for mental health, including web applications or smartphone apps, and to investigate attitudes toward using a mental health assessment app to screen, aid in the diagnosis, and triage mental health concerns, respectively. The findings from this survey have important implications regarding the mental health care experiences of those who sought help and their preferences for future mental health app design.

## Methods

### Participants

Participants were recruited between November 15, 2021, and February 7, 2022, via email, paid Facebook and Instagram advertisements, unpaid posts on the Cambridge Centre for Neuropsychiatric Research Facebook and Twitter pages, and Reddit. Recruitment messages were also disseminated by word of mouth and through relevant foundations and support groups. The inclusion criteria for the study were as follows: (1) aged ≥18 years, (2) UK residence, and (3) had visited an HCP in the previous 5 years (between 2016 and 2021) to discuss mental health symptoms. These criteria were chosen to yield an adult population sample that would represent potential users of a mental health app. The 5-year time frame was chosen to gather feedback and experiences regarding recent mental health provision provided via health care services and workplaces in the United Kingdom. There were no other inclusion criteria. Participants were invited to enter their email address for the chance to win 1 of 3 high street vouchers worth £50 (US $60) each.

### Ethics Approval

The study was approved by the Cambridge Human Biology Research Ethics Committee (PRE.2021.053).

### Informed Consent

All participants provided informed consent electronically before participating in the study.

### Materials and Procedure

An anonymous web-based survey was created using Qualtrics XM to explore the current state of mental health care provision in the United Kingdom and attitudes toward using a mental health assessment app to screen, diagnose, and triage mental health symptoms. The survey could be completed in 20 to 25 minutes and comprised four sections: (1) sociodemographic information, (2) mental health symptoms and experience of health care service provision, (3) the impact of mental health symptoms in the workplace as well as the experience of mental health provision in the workplace, and (4) experiences and interest in using digital technology for mental health. The survey was adaptive in nature such that only relevant questions were asked based on previous responses. Questions regarding waiting times for diagnoses, therapy, or counseling refer to the time involved in waiting from the first contact with an HCP or initial referral or self-referral to receiving therapy or counseling services.

### Patient and Public Involvement Review

Throughout the planning stage of this study, we engaged in various patient and public involvement (PPI) activities to thoroughly review the study’s methods and materials. More specifically, the web-based survey, the information sheet, consent form, and study debrief were first designed with input from an experienced psychiatrist (SB) and then reviewed in consultation with the members of the Cambridge University Hospitals (CUH) PPI panel. The PPI review strategy was designed collaboratively with the PPI engagement and communications lead and the PPI engagement coordinator of the CUH PPI panel. First, the survey and study materials were reviewed by the 3 PPI panel members with lived experience of mental health concerns in one-on-one Zoom (Zoom Video Communications, Inc) sessions with 2 authors (BS and NAM-K), with each session lasting approximately 1 hour. Second, the reviewed versions of the survey and study materials were further reviewed independently in both their Word (Microsoft Corp) and web-based formats by other members of the CUH PPI panel. All feedback was collated in a comprehensive report, and changes were made to the survey and study materials accordingly. Overall, the clarity of the survey and study materials was improved through careful adjustments to the wording. The survey was shortened, and questions were optimized to ease completion.

### Data Analytic Strategy

Quantitative data (ie, frequencies and percentages) were analyzed and processed in Excel (version 2206; Microsoft Corp). Figure S1 in Multimedia Appendix 1 was created in R (version 4.1.2; R Foundation for Statistical Computing), using the packages *rgdal* (version 1.6-2), *gpclib* (version 1.5-6), and *maptools* (version 1.1-5) for spatial data manipulation and *ggplot2* (version 3.4.0) for plotting. Data included sociodemographic information, mental health characteristics, mental health provision, and the effects of mental health on work life, as well as experience in the use of digital technologies for mental health and interest in using a mental health assessment app. Given that the survey was adaptive in nature, such that only relevant questions were asked based on previous responses, sample numbers varied per question, with percentages calculated using the total *n* per question rather than the overall sample size. Where the *n* differed from the overall sample size, this has been highlighted in the tables.

## Results

### Sociodemographic Characteristics

A total of 1072 participants commenced the survey, of whom 618 (57.65%) confirmed that they had discussed their mental health with an HCP in the previous 5 years and answered at least the first 2 sections of the survey. The responses from these 618 participants were analyzed.

A summary of the respondents’ sociodemographic information can be found in Table 1. The average age of the respondents was 49.24 (SD 14.89) years, and the majority were women (437/618, 70.7%), White (595/618, 96.3%), and native English speakers (596/618, 96.4%). Of the 618 participants, 232 (37.5%) had at least an undergraduate degree, 193 (31.2%) were single, and 191 (30.9%) were married or in a civil partnership. Regarding accommodation characteristics, living alone or with a partner was the most common arrangement, with 35.6% (220/618) living alone, 26.7% (165/618) living with a partner, and 16.7% (103/618) living with a partner and children. Of the 618 participants, 266 (43%) were currently employed (ie, full time, part time, or self-employment) and 392 (63.4%) had an annual household income of <£35,001 (US $42,001) before tax. The survey was completed from across the United Kingdom. The distribution and frequency of post code areas can be seen in Figure S1 in Multimedia Appendix 1. Participants came from 111 of the main 125 UK postcode areas.

**Table 1:** Sociodemographic characteristics (n=618).

Sociodemographic characteristics			Values
Age (years), mean (SD)			49.24 (14.89)
**Gender, n (%)**			
	Woman	437 (70.7)	
	Man	158 (25.6)	
	Nonbinary	14 (2.3)	
	Prefer not to answer	9 (1.5)	
**Ethnicity, n (%)**			
	Asian or Asian British	6 (1)	
	Black, African, Caribbean, or Black British	4 (0.7)	
	White	595 (96.3)	
	Mixed or multiple ethnic groups	8 (1.3)	
	Other	5 (0.8)	
**Language or native tongue, n (%)**			
	English	596 (96.4)	
	Other	22 (3.6)	
**Education, n (%)**			
	Below GCSE^a^ or equivalent	83 (13.4)	
	GCSE or equivalent	126 (20.4)	
	A level or IB^b^ or advanced higher	141 (22.8)	
	Undergraduate degree	133 (21.5)	
	Postgraduate degree	99 (16)	
	Prefer not to answer	36 (5.8)	
**Relationship status, n (%)**			
	Single	193 (31.2)	
	Married or civil partnership	191 (30.9)	
	Cohabiting	75 (12.1)	
	Separated	25 (4.1)	
	Divorced	80 (12.9)	
	Widowed	28 (4.5)	
	Other	16 (2.6)	
	Prefer not to answer	10 (1.62)	
**Living arrangement, n (%)**			
	Living alone	220 (35.6)	
	Living in shared accommodation with previously unknown individual or individuals	24 (3.9)	
	Living with friends	21 (3.4)	
	Living with relatives, including single parent	85 (13.8)	
	Living with a partner	165 (26.7)	
	Living with a partner and children	103 (16.7)	
**Employment status^c^, n (%)**			
	Full-time employment	154 (24.9)	
	Part-time employment	78 (12.6)	
	Self-employment	34 (5.5)	
	Parental leave or care for a family member	10 (1.6)	
	Student	31 (5)	
	Voluntary work	32 (5.2)	
	Retired	117 (18.9)	
	Unemployed	146 (23.6)	
	Other	68 (11)	
	Prefer not to answer	13 (2.1)	
**Household income (£; £1=US $1.2), n (%)**			
	<15,000	205 (33.2)	
	15,001-25,000	119 (19.3)	
	25,001-35,000	68 (11)	
	35,001-45,000	40 (6.5)	
	45,001-55,000	32 (5.2)	
	55,001-65,000	21 (3.4)	
	65,001-75,000	6 (1)	
	75,001-85,000	11 (1.8)	
	>85,001	23 (3.7)	
	Prefer not to answer	93 (15.1)	

^a^GCSE: General Certificate of Secondary Education.

^b^IB: International Baccalaureate.

^c^Percentages add up to >100% because respondents could select multiple options.

### Mental Health Symptoms and Diagnosis Provision

A summary of the respondents’ mental health symptoms and diagnosis provision can be found in Table S1 in Multimedia Appendix 1 and Table 2. The majority of the respondents (555/618, 89.8%) had discussed their mental health with a general practitioner (GP) in the last 5 years, with 58.4% (361/618) having also seen a therapist or counselor and 34.6% (214/618) having also seen a psychiatrist. Mental health care visits were typically provided free of charge via the National Health Service (NHS; 571/618, 92.4%). Of the 618 respondents, 231 (37.4%) had been diagnosed with a mental health disorder since 2016. Participants were most commonly diagnosed with depression (165/231, 71.4%) and generalized anxiety disorder (126/231, 54.5%).

Diagnostic waiting times from first contact with an HCP varied across diagnoses, with diagnoses of neurodevelopmental disorders and bipolar disorder having the highest proportions of waiting times over 6 months (Table 2). More specifically, 54% (30/56) of the neurodevelopmental disorder diagnoses, 48% (25/52) of the bipolar disorder diagnoses, and 47.5% (48/101) of the personality disorder diagnoses were received after waiting for >6 months from first contact with an HCP. Of the participants (353/503, 70.2%) who waited >1 month to receive a diagnosis, 74.2% (262/353) stated that this resulted in their symptoms worsening, and 47% (166/353) also stated that their day-to-day life was made harder by the wait. Another common consequence of the waiting period was seeking emergency mental health care (109/353, 30.9%).

**Table 2:** Distribution of waiting times per diagnosis (n=581)^a^.

Diagnosis	Diagnoses, n (%)				
	>1 month	Between 1 and 3 months	Between 3 and 6 months	<6 months	Not sure
Depression (n=411)	155 (37.7)	74 (18)	66 (16.1)	55 (13.4)	61 (14.8)
Bipolar disorder (n=52)	5 (9.6)	9 (17.3)	1 (1.9)	25 (48.1)	12 (23.1)
GAD^b^ (n=263)	84 (31.9)	56 (21.3)	42 (16)	41 (15.6)	40 (15.2)
Social anxiety or phobia (n=137)	27 (19.7)	27 (19.7)	28 (20.4)	35 (25.6)	20 (14.6)
Panic disorder or panic attacks (n=132)	36 (27.3)	24 (18.2)	25 (18.9)	24 (18.2)	23 (17.4)
OCD^c^ (n=53)	6 (11.3)	9 (17)	15 (28.3)	13 (24.5)	10 (18.9)
Insomnia or another sleep disorder (n=62)	13 (21)	9 (14.5)	17 (27.4)	10 (16.1)	13 (21)
Schizophrenia or psychosis (n=34)	12 (35.3)	2 (5.9)	9 (26.5)	7 (20.6)	4 (11.8)
Personality disorders (n=101)	10 (9.9)	11 (10.9)	13 (12.9)	48 (47.5)	19 (18.8)
Eating disorders (n=38)	7 (18.4)	4 (10.5)	5 (13.2)	15 (39.5)	7 (18.4)
PTSD^d^ or a trauma-related mental health disorder (n=152)	25 (16.4)	21 (13.8)	32 (21.1)	58 (38.2)	16 (10.5)
A neurodevelopmental disorder (eg, ASD^e^, ADHD^f^, and learning disability; n=56)	5 (8.9)	7 (12.5)	8 (14.3)	30 (53.6)	66 (10.7)
Other disorder or disorders (n=46)	9 (19.6)	2 (4.4)	4 (8.7)	19 (41.3)	12 (26.1)

^a^The overall n and n per diagnosis add up to different numbers than if you were to add diagnoses per condition before 2016 and after 2016, as some participants reselected the same diagnosis along with different ones for before 2016 and after 2016.

^b^GAD: generalized anxiety disorder.

^c^OCD: obsessive compulsive disorder.

^d^PTSD: posttraumatic stress disorder.

^e^ASD: autism spectrum disorder.

^f^ADHD: attention-deficit/hyperactivity disorder.

### Mental Health Support and Treatment Provision

A summary of the respondents’ mental health support and treatment provision can be found in Table S1 in Multimedia Appendix 1. Of the 618 respondents, 386 (62.5%) stated that they felt that their mental health symptoms were not always taken seriously by their HCP, with 297 (48.1%) reporting receiving no information about mental health disorders (eg, what the symptoms are, how common they are, why they may arise, and treatment options). Similarly, 77.5% (461/595) felt that they were not always given the opportunity to discuss mental health support and treatment options related to the management of their mental health symptoms. The most common treatment options were medication (517/618, 83.7%) and counseling or psychotherapy (481/618, 77.8%), with 50.6% (301/595) finding treatment moderately or extremely helpful.

Of those who received counseling or psychotherapy, 41.8% (201/481) reported having to wait >3 months from referral or self-referral, with the majority of respondents (212/334, 63.5%) who waited >1 month stating that this had resulted in their symptoms getting worse. Of the 481 respondents, 213 (44.3%) were referred for counseling or therapy by an HCP, whereas 209 (43.5%) had self-referred. Of the 209 respondents who had self-referred, 159 (76.1%) stated that the self-referral process had been easy. Of the 50 respondents who found it at least slightly difficult, long waiting times (n=37, 74%) and not knowing how to choose a counseling service or a therapist (n=22, 44%) were the main barriers to self-referral.

Critically, of the 96 respondents who did not find any treatment or support (eg, medication, counseling, or psychotherapy) at all helpful, 76 (79%) stated that the treatment made them feel worse, with 63% (n=48) having discontinued treatment and not having found an effective treatment or support for their mental health symptoms at the time of completing the survey.

### Mental Health in the Workplace

A summary of the respondents’ mental health in the workplace can be found in Table S1 in Multimedia Appendix 1. Of the 290 respondents who were employed (full time or part time) or self-employed, on parental leave, or doing voluntary work, 256 (88.3%) reported that their mental health symptoms had affected their experience in the workplace. Of these 256 respondents, 226 (88.3%) stated that their mental health symptoms had increased their stress levels at work. This was followed by decreased productivity (145/256, 56.6%) and having an impact on work relationships (145/256, 56.6%). The most common sources of mental health support provided by employers were wellness programs (89/263, 33.8%) and training courses or support related to workplace mental health (86/263, 32.7%). When respondents who were employed full time or part time, on parental leave, or doing voluntary work were asked what kind of mental health services they would like to receive from their workplace, most of them (125/262, 47.5%) stated that they would be interested in training courses or support, followed by an interest in mental health app provision (110/262, 41.8%) and wellness programs (108/262, 41.1%).

### Experiences and Interest in Using Digital Technology for Mental Health

A summary of the respondents’ experiences and interest in using digital technology for mental health can be found in Table S2 in Multimedia Appendix 1.

The majority of the respondents (545/617, 88.3%) sought help and information on the web regarding their mental health symptoms, with the information most commonly searched for being symptom characteristics (446/543, 82.1%) and treatment or therapy options (390/543, 71.8%). Of the 612 respondents, 272 (44.4%) had used a web application or smartphone app for their mental health, with 206 (75.5%) stating that they did not have to pay to use these apps. Of the 582 respondents, 176 (30.2%) stated that they were using wearable devices (eg, a smartwatch), with the most common uses being tracking physical activity (149/176, 84.7%) and heart rate (111/176, 63.1%), along with monitoring sleep (106/176, 60.2%).

When the respondents were asked to select features for their ideal mental health app, the feature most desired was psychoeducation (ie, gaining a better understanding of one’s mental health state; 364/596, 61.1%). This was followed by receiving help to be referred to an HCP (332/596, 55.7%) and monitoring of symptoms over time (314/596, 52.7%). Of the 594 respondents, 318 (53.5%) stated that being able to search for, and use, the app independently of HCPs would encourage them to use it, whereas 250 (42.1%) stated that referral to the app by an HCP would be a prompt to use the app. Of the 593 respondents, 389 (65.6%) reported that they would prefer to use a web application (ie, available either using a computer or a mobile phone), whereas 204 (34.4%) preferred a smartphone-only app. Of the 591 respondents, 299 (50.6%) were Android mobile phone users, closely followed by 246 (41.6%) iPhone users. The most common browsers used by respondents to search the web were Google Chrome (343/590, 58.1%) and Safari (133/590, 22.5%).

When the respondents were asked about their hypothetical interest in using an app to complete a mental health assessment before an appointment with an HCP, only 6.8% (40/590) said that they would not be interested in using the app. Of the 550 respondents who were interested, 289 (52.5%) expressed a preference for the results of the mental health assessment to be sent directly to their health care provider, whereas 186 (33.8%) preferred taking their mental health assessment results independently to an HCP. Regarding a hypothetical results report, respondents were most interested to receive an overall severity score of their mental health symptoms (441/546, 80.8%) and an indication of whether they should get mental health support (454/546, 83.2%). Of the 545 respondents, 417 (76.5%) stated that they would seek professional help if the results from the mental health assessment suggested that they may be experiencing a mental health disorder, with the majority being most likely to see their GP (323/523, 61.8%). Of the 545 respondents, 106 (19.5%) stated that they would want their mental health assessment results to be reviewed by a psychiatrist, even if it came at a cost, whereas 256 (47%) were not interested at all in the review, and 183 (33.6%) were not sure. Most of the respondents (301/542, 69.6%) stated that they would feel at least slightly comfortable if the mental health assessment app used artificial intelligence (AI) to assess mental health symptoms. Of the 575 respondents, 415 (72.2%) stated that they would not be willing to pay for the mental health assessment app.

## Discussion

### Principal Findings and Comparison With Prior Work

The aim of this study was to investigate the current state of mental health provision in the United Kingdom as well as understand the utility of, and interest in, digital mental health technologies. To do this, we implemented an anonymous web-based survey comprising four sections: (1) sociodemographic information, (2) mental health symptoms and experience of health care service provision, (3) impact of mental health symptoms in the workplace and experience of mental health provision in the workplace, and (4) experiences and interest in using digital technology for mental health. Overall, the findings from this survey study support the view that there are critical gaps in current mental health care provision in the United Kingdom.

The survey results support the central role of GPs in the delivery of mental health care. Indeed, out of the various HCPs involved in the delivery of mental health care in the United Kingdom, GPs were consulted by 89.8% (555/618) of the respondents. Previous evidence cites mental health problems as the second most common reason for primary care consultations in the United Kingdom, and GPs reportedly spend approximately 30% of their time on mental health concerns [16]. Critically, the United Kingdom is experiencing a GP retention crisis: GPs are overwhelmed with an increasing workload and administrative burden, often leading to time-pressured consultations that can affect the quality of care [17]. In addition, in some instances, GPs have reported that their practices were underprepared to provide timely and effective mental health support [18]. This should be considered when considering the gaps and possible improvement strategies regarding the delivery of mental health support.

In addition to the key role of GPs, our findings identify prolonged waiting times for diagnosis and treatment of mental health disorders as a significant area for improvement. A total of 70.2% (353/503) of our respondents waited >4 weeks to receive a diagnosis, and 41.8% (201/481) had to wait >12 weeks to begin counseling or psychotherapy. We observed variation in the waiting times of different diagnoses. In our sample, diagnoses of neurodevelopmental disorders (eg, autism spectrum disorder and attention-deficit/hyperactivity disorder), bipolar disorder, and personality disorders took the longest to diagnose. These findings are in line with well-documented evidence; for instance, the diagnosis of bipolar disorder is frequently delayed owing to a variety of factors, including time constraints during consultations in primary care settings coupled with the underreporting of manic or hypomanic symptoms and symptom overlap with other conditions, especially major depressive disorder [19]. Numerous studies and systematic reviews report that >40% of patients with bipolar disorder were initially misdiagnosed with unipolar depression [20-23]. Less well documented but still acknowledged is the fact that the diagnosis of personality disorders remains challenging, with case reports discussing delays and frequent misdiagnoses. Similarly, research has shown there are significant rates of misdiagnosis for neurodevelopmental disorders such as attention-deficit/hyperactivity disorder and autism spectrum disorder, especially in the adult female population [24,25].

Interestingly, our results show that some conditions, including unipolar depression (ie, major depressive disorder; 229/411, 55.7%) and generalized anxiety disorder (140/263, 53.2%), were mostly diagnosed within <3 months. Although we did not specifically ask which HCP provided the diagnosis, the relatively short time frame suggests that these patients were diagnosed in a primary care setting because waiting times for specialist secondary care assessments are considerably longer in the United Kingdom [26]. The possibility of accessing timely mental health support at the primary care level is very important. However, evidence suggests a high rate of misdiagnoses in primary care not only for more complex psychiatric disorders such as bipolar disorder but also for other mental disorders. A large meta-analysis of >100 studies involving 50,371 patients showed that GPs correctly identified unipolar depression in 47% of the cases, with misidentification outnumbering missed cases [27]. Similarly, it is estimated that 47% of anxiety disorders are not recognized in primary care, and two-thirds are misdiagnosed with other mental health conditions and hence do not receive the most appropriate treatment [28]. This can also be due to the fact that patients with mental health concerns frequently present with psychosomatic symptoms [29].

This evidence should not be interpreted as a criticism of primary care support but rather as a call for supporting nonspecialists in making more accurate triage decisions and diagnoses [27]. Time pressure, comorbidities, and a tendency to prioritize physical health over mental health are all aspects that contribute to the misdiagnosing of patients with mental health conditions in primary care [27]. Importantly, delays and misdiagnoses have well-known negative impacts on health at both the individual and population levels. Especially in the case of bipolar disorder, a misdiagnosis can have particularly detrimental consequences, with patients being placed at a significantly higher risk of suicide, treatment resistance, and manic episodes if they are treated with antidepressants instead of mood stabilizers. This highlights the importance of undertaking timely, comprehensive, and differential diagnostic mental health assessments.

In our study, participants reported that waiting times had detrimental effects on their mental health symptoms and quality of life. This mirrors recent findings from the Royal College of Psychiatrists reporting that 39% of patients saw a deterioration in their mental health while waiting for mental health support, leading to lower quality of life (89%); relationship problems (33%); financial troubles (30%); and problems at work, including job losses (18%) [26]. Critically, this study shows that prolonged mental health assessment timelines led 30.9% (109/353) of the respondents to seek emergency care. Once again, this is not an isolated finding because the Royal College of Psychiatrists reported that 40% of the patients waiting for mental health treatment contact emergency or crisis services, with 1 in 9 ending up in the emergency room [26].

Thus, the evidence gathered in this study, viewed in the broader context of related research, supports the introduction of a mental health assessment tool that could act as a triage tool for nonspecialists and specialist mental health care providers. If delivered digitally before the physician’s appointment, the tool could gather and summarize symptom and personal information, thus providing HCPs with diagnostically relevant information before an in-person assessment. This may relieve time pressure and improve accuracy.

Importantly, a substantial proportion of the respondents stated that they felt that their mental health symptoms were not always taken seriously by their health care provider (386/618, 62.5%) and that they were not given any information about mental health disorders (eg, what the symptoms are, how common they are, and why they may arise) or about treatment and psychotherapy options (297/618, 48.1%). This constitutes a concerning finding because the National Institute for Health and Care Excellence (NICE) guidelines recommend that symptoms and treatment options should be explained and discussed with the patient; for instance, in the case of depression, the guidance’s *principle of care* section specifically states that the clinician should discuss the nature of the condition, the symptoms, the treatment options, and the steps to possible recovery, as well as “taking steps to create an optimistic and welcoming atmosphere to reduce stigma” [30]. Unfortunately, the understaffed and time-pressured environment in which HCPs operate often makes adherence to the NICE guidelines close to impossible.

In addition to not being given sufficient information, 16.1% (96/595) of the respondents were not offered treatment or support (eg, medication, counseling, or psychotherapy) that they found helpful, with the majority (76/90, 79%) stating that the treatment made them feel worse; consequently, 63% (48/76) of the respondents had discontinued treatment and not found an effective alternative. However, it was reassuring to find that the majority of those who had self-referred to psychotherapy or counseling found the self-referral process easy (159/209, 76.1%). For those who found it difficult (50/209, 23.9%), once again it was the long waiting times that were perceived as a barrier (37/50, 74%), followed by not knowing how to choose a counseling service or a psychotherapist (22/50, 44%); hence, more effective signposting and guidance are needed. Interestingly, the perceived lack of mental health information and referral signposting were mirrored in the most desired features for a mental health app chosen by our survey participants (ie, psychoeducation and referral to an HCP), which are discussed in greater detail in the following paragraphs.

Finally, the impact of mental health symptoms on work activities cannot be overstated. Of the 290 respondents in employment, 256 (88.3%) disclosed that their mental health symptoms had a negative impact on their work experience. Indeed, although mental health concerns are acknowledged in the United Kingdom, the stigma and social exclusion of those with mental health symptoms often translate into poor mental health awareness and support in the workplace [31]. Critically, mental health disorders, representing approximately one-fifth of the burden of adult disease in the United Kingdom, play a significant role in sickness absence from work [32,33]. In turn, absence owing to mental health symptoms can lead to decreased workplace well-being and deteriorating work relationships, in addition to productivity loss, with negative economic outcomes for both employers and wider society.

Thus, there have been research efforts in designing various types of eHealth interventions (eg, mindfulness apps, web-based cognitive behavioral therapy, and stress management apps) to reduce mental health burden in the workplace. However, these interventions are often not tailored to any specific disorder, the evidence from systematic reviews and meta-analyses is inconclusive, and efficacy as well as employee acceptability are highly variable [34,35]. In addition, eHealth interventions are often designed to equip employees with generic coping strategies rather than identifying and treating diagnostically relevant symptoms. Considering that individuals who are later diagnosed with a mental health disorder tend to visit GPs more frequently and, before their diagnosis, take more sick leave [36], greater efforts should be focused on providing the means to identify mental health concerns in the workplace early.

This call to action echoes the occupational mental health guidance newly released by the Royal College of Psychiatrists, which calls for better support for people with mental health problems to find, return to, and remain in work [37]. A study found that the introduction of a workplace intervention in the form of a screening and care management for those living with (or at risk of) depression was estimated to cost £31 (US $37.2) per employee for assessment and £240 (US $288) for therapy and could yield a 300% return on investment over a 2-year period [6]. In this study, respondents seemed open to receiving support from their workplace, with mental health apps being one of the desired means of support. According to previous evidence, the introduction of digital health technologies in the workplace has often been welcomed, with caution and concerns around health data sharing, privacy, and autonomy [38,39].

In the context of digital mental health assessments, any symptom data disclosed by an individual are to be considered health data under the General Data Protection Regulation (GDPR) and thus must be handled accordingly as per the regulation, that is, the individual should be able to access all personal data and outcomes of sensor and intervention technologies without the interference of others, and their employer should not have access to any of these data or outcomes or be able to derive these outcomes from group data [39]. These crucial data protection points must be addressed during the development phase of any digital health tool as well as in its implementation and presentation to future users. Overall, we report a general interest in digital mental health tools offered by employers; however, further research is required to better characterize the acceptability of a mental health assessment app in workplace settings.

Regarding the use of digital technologies for mental health, our findings should be interpreted keeping the following population characteristics in mind: (1) of the 617 respondents, 545 (88.3%) had looked up information on the web regarding their mental health symptoms and treatment or therapy options; (2) two-fifths (272/612, 44.4%) of the respondents had used a web application or smartphone app for their mental health; and (3) of the 582 respondents, 176 (30.2%) had also used wearable devices such as smartwatches to track their physical well-being and sleep. Various studies have found that access to, and the use of, digital technologies, including smartphones, web-based programs, and social media, among individuals living with mental illness is increasing, with well-documented evidence arising from various clinical and community settings [40-43]. Thus, although potentially inflated by recruitment bias, these findings, which are in line with previous evidence, depict the prevalence and use of digital technology for mental health.

The responses in our study offered valuable insights into users’ preferred mental health app features, with psychoeducation (ie, gaining a better understanding of one’s mental health status), symptom monitoring, and referrals to HCPs being the most popular. These findings may be the result of the perceived lack of provision of mental health information discussed previously and the lack of guidance on where and how to choose counseling and support options reported by those who went through a self-referral process. In addition, the focus on psychoeducation and symptom monitoring stresses the importance and potential perceived benefit of empowering patients with evidence-based knowledge and a sense of control over their condition. Crucially, providing psychoeducation can increase help-seeking behaviors, reduce stigma, and improve patient engagement and adherence to the HCPs’ recommendations [44-46].

Furthermore, psychoeducation can increase mental health literacy [47], and, if supplemented with self-help tips, it can improve coping self-efficacy (ie, ability to cope with distress and adversity) [48]. Along similar lines, self-monitoring is thought to improve mental health and well-being by increasing emotional self-awareness (ie, the individual’s ability to identify their symptoms in case of a relapse). Thus, a combination of the desired features as selected by the respondents could offer multiple benefits in managing mental health conditions while addressing some of the perceived gaps in mental health care provision. Implementing the desired features discussed here may also offer benefits beyond the individual patient. Evidence in GP practices has demonstrated that providing self-care advice on GP websites and active signposting to relevant nonmedical staff (ie, social prescribers) reduces the number of inappropriate GP appointments, with GPs reporting being able to spend more time with patients. In addition, with patients receiving self-care information from GPs, they became better informed of their choices and their support options beyond the GP [49].

When asked about a hypothetical mental health assessment app, 93.2% (550/590) of the respondents reported that they would like to try it out and offered insights into how they would like to receive results and follow-up contact from an HCP. A severity score and actionable help seeking or triage recommendations were the most requested features for the mental health assessment results report. Regarding the preferences of sharing the results report with an HCP, one-third (186/550, 33.8%) of the respondents reported preferring to independently decide whether to share their results. Critically, 19.6% (107/545) of the respondents said that they were unsure about seeking help if the results report told them that they may be experiencing a mental health disorder. This result has important development implications. It suggests that there may be differences in users’ behaviors upon receipt of their mental health assessment results, with some users wanting to seek help and others preferring not to do so. The latter behavior may be due to stigmatizing beliefs, perceived difficulties in expressing mental health concerns, a preference for self-reliance, or perhaps previous negative health care experiences, which are barriers identified in previous research [50]. Considering this evidence, the importance of providing clear psychoeducation, self-help tips, and guidance on how to seek help cannot be overstated. In addition, our results call for future research on the barriers and facilitators to help seeking in the context of delivering a digital mental health assessment.

Despite the interest in the digital mental health assessment app, willingness to pay was low with 72.2% (415/575) of the respondents stating that they would not be willing to pay for it. This may be due to a variety of reasons; for instance, 75.7% (206/272) of our app-using respondents were users of free apps. Critically, willingness to pay for a health app is often dependent on the perceived benefits derived from using the app [51]. On the basis of our respondents’ preferences, receiving a diagnosis was not among the most desirable features in a mental health app; hence, the perceived value of a mental health assessment app may not have been as high as that of an *ideal* app that offers psychoeducation, symptom monitoring, and HCP referral information and pathways, alongside an assessment of symptoms.

Moreover, the British population benefits from a national health care system, the NHS, that provides most mental health (and physical) care services for free; thus, most of the respondents may expect the digital mental health assessment to be provided through the NHS at no out-of-pocket cost. Similarly, only a small proportion of the respondents (106/545, 19.4%) were interested in a potentially costly psychiatrist review of their mental health assessment results report. Interestingly, most of the respondents (377/542, 69.6%) stated that they would feel at least slightly comfortable if the mental health assessment app used AI to assess mental health symptoms. This is in line with previous evidence that showed moderate acceptability (60%-80%) of AI-led chatbots in general care as well as mental health care [52,53]. Hesitancy toward AI is known to be exacerbated by a variety of factors, including poorer perceived digital skills and a dislike for digital interactions, but this can be mitigated by perceived utility and trustworthiness [53]. Hence, further research is required to more deeply investigate the impact that AI could have on app uptake and any strategies that could improve hesitant users’ perspectives. Overall, all discussed findings relating to app features, potential implementation pathways, and app technology aspects have important implications in the design and funding of a viable and accessible mental health assessment app.

### Limitations

The survey was comprehensive and carefully designed with inputs from an experienced psychiatrist as well as from individuals with lived experience of mental health concerns. However, most of the individuals who participated in this study were women (437/618, 70.7%), more highly educated than average (232/618, 37.5%) had at least an undergraduate degree), and spoke English as their first language (596/618, 96.4%). Thus, the findings from this study may not be fully generalizable to the broader British population. Notably, 34.6% (214/618) of the respondents had also seen a psychiatrist, which suggests that the survey had reached a significant proportion of individuals who likely experienced severe mental health symptoms that required psychiatric support or assessment. In addition, this survey may have been subject to recruitment bias because individuals with negative experiences of mental health care provision may have been more receptive to the recruitment and more likely to enroll in the study. Less than half (272/612, 44.4%) of the respondents had used a mental health app before; hence, the interest in trying out a mental health assessment app cannot be explained only by habit or recruitment bias.

### Conclusions

The findings from this study highlight the need to improve the early diagnosis of mental health disorders, especially bipolar disorder and neurodevelopmental disorders. This could have several benefits, including higher patient satisfaction and well-being as well as decreased mental health care costs. However, the implementation of a mental health assessment app has the potential to create a bottleneck effect in which assessment and diagnostic waiting times are shortened, but access to support remains limited owing to a lack of resources. Thus, it is fundamental to provide in-app features (eg, psychoeducation, symptom monitoring, and triaging and sources of support) to assist patients through the waiting times and their care journeys. These features also have the potential to increase mental health literacy, coping strategies, and symptom awareness, which is likely to improve mental health outcomes. Finally, funding strategies and choice of technology (to use AI or not to use AI?) are important aspects to consider in the future development of the digital mental health assessment tool; critically, they may have an impact on the accessibility dictated by purchasing power and digital literacy with the risk of excluding various groups of individuals who may benefit from the app.

## Appendix

Multimedia Appendix 1 Postcode area map of survey participants, their mental health symptoms and their experiences of health care service provision, and their experiences and interest in using digital technology for mental health.

## Abbreviations

AI: artificial intelligence
CUH: Cambridge University Hospitals
GDPR: General Data Protection Regulation
GP: general practitioner
HCP: health care professional
NHS: National Health Service
NICE: National Institute for Health and Care Excellence
PPI: patient and public involvement

## Data Availability

The data that support the findings of this study are available from the corresponding author (SB) upon reasonable request.
