# Supplementary material for: Opportunities for the Implementation of a Digital Mental Health Assessment Tool in the United Kingdom: Exploratory Survey Study
Source: JMIR Form Res. 2023 Aug 7;7:e43271. doi: 10.2196/43271 (PMC10442733; doi:10.2196/43271)
Supplement: Multimedia Appendix 1 [file formative_v7i1e43271_app1.docx]

**Multimedia Appendix 1.**


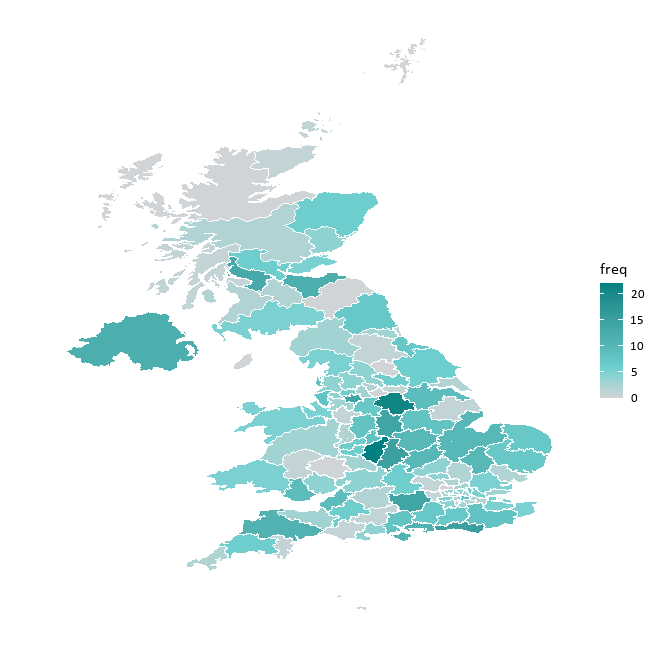
 **Figure S1.** Postcode area map of survey participants (n=611, 7 data points did not correspond to any area and were disregarded).

**Table S1.** Mental health symptoms and experience of health care service provision (N=618).

|  | | ***n* (%)** |
| --- | --- | --- |
| HCPs visited in last 5 years^a^ | GP | 555 (89.81) |
|  | Mental health link worker | 143 (23.14) |
|  | Mental health nurse | 241 (39.00) |
|  | Psychiatrist | 214 (34.63) |
|  | Psychologist | 172 (27.83) |
|  | Therapist (eg, a counsellor) | 361 (58.41) |
|  | Other | 50 (8.09) |
| Types of health care visits^a^ | Free-of-charge services via the NHS | 571 (92.39) |
|  | Free-of-charge services via a charity or a not-for-profit organisation (eg, Mind, Rethink) | 179 (28.96) |
|  | Private healthcare services covered by private medical insurance | 24 (3.88) |
|  | Private healthcare services which you had to pay yourself | 118 (19.09) |
|  | Other | 41 (6.63) |
| Assessment and diagnosis status^a^ | Yes, before 2016 | 350 (56.63) |
|  | Yes, after 2016 | 231 (37.38) |
|  | No, I was assessed and I was told I don’t have a mental health disorder | 23 (3.72) |
|  | No, but I am on a waiting list to be assessed | 19 (3.07) |
|  | No, I was never assessed and I am not on a waiting list to be assessed | 82 (13.27) |
| Diagnoses before 2016 (n=350)^a^ | Depression | 300 (85.71) |
|  | Bipolar disorder | 40 (11.43) |
|  | Generalized anxiety disorder | 167 (47.71) |
|  | Social anxiety or phobia | 95 (27.14) |
|  | Panic disorder or panic attacks | 91 (26.00) |
|  | Obsessive-compulsive disorder | 36 (10.29) |
|  | Insomnia or another sleep disorder | 43 (12.29) |
|  | Schizophrenia or psychosis | 25 (7.14) |
|  | A personality disorder | 62 (17.71) |
|  | An eating disorder | 20 (5.71) |
|  | Post-traumatic stress disorder or a trauma-related disorder | 92 (26.29) |
|  | A neurodevelopmental disorder (eg, autism spectrum disorder, attention deficit hyperactivity disorder, learning or intellectual disability) | 23 (6.57) |
|  | Other | 30 (8.57) |
|  | I am not sure | 10 (2.86) |
| Diagnoses after 2016 (n=231)^a^ | Depression | 165 (71.43) |
|  | Bipolar disorder | 13 (5.63) |
|  | Generalized anxiety disorder | 126 (54.55) |
|  | Social anxiety or phobia | 51 (22.08) |
|  | Panic disorder or panic attacks | 50 (21.65) |
|  | Obsessive-compulsive disorder | 19 (8.23) |
|  | Insomnia or another sleep disorder | 21 (9.09) |
|  | Schizophrenia or psychosis | 11 (4.76) |
|  | A personality disorder | 42 (18.18) |
|  | An eating disorder | 21 (9.09) |
|  | Post-traumatic stress disorder or a trauma-related disorder | 69 (29.87) |
|  | A neurodevelopmental disorder (eg, autism spectrum disorder, attention deficit hyperactivity disorder, learning or intellectual disability) | 35 (15.15) |
|  | Other | 19 (8.23) |
|  | I am not sure | 4 (1.73) |
| Effects of waiting time for diagnosis (>1 month) (n=353)^a^ | Symptoms got worse | 262 (74.22) |
|  | Seeking emergency mental health care | 109 (30.88) |
|  | Made day-to-day harder | 166 (47.03) |
|  | Symptoms stayed the same | 50 (14.16) |
|  | Symptoms got better | 6 (1.70) |
|  | Other | 52 (14.73) |
| Symptoms taken seriously by HCP | Yes | 232 (37.54) |
|  | No | 102 (16.50) |
|  | Mixed experience | 284 (45.95) |
| Given information about mental health symptoms by HCP | Yes, without me asking for it | 214 (34.63) |
|  | Yes, but had to specifically ask for it | 107 (17.31) |
|  | No | 297 (48.06) |
| Type of support received^a^ | Medication | 517 (83.66) |
|  | Counselling or therapy | 481 (77.83) |
|  | Other | 110 (17.80) |
|  | None | 33 (5.34) |
| Treatment helpfulness (n=595) | Not at all | 96 (16.13) |
|  | Slightly | 198 (33.28) |
|  | Moderately | 225 (37.82) |
|  | Extremely | 76 (12.77) |
| Unhelpful treatment made symptoms worse (n=96) | Yes | 76 (79.17) |
|  | No | 20 (20.83) |
| Unhelpful treatment discontinuation (n=76) | Yes, and I still have not found treatment or support that works | 48 (63.16) |
|  | Yes, and I have found a different treatment or support that works | 8 (10.53) |
|  | No, I have continued with the same treatment | 20 (26.32) |
| Opportunity to discuss treatment or support options (n=595) | Yes, always | 111 (18.66) |
|  | Sometimes | 320 (53.78) |
|  | No, never | 141 (23.70) |
|  | I am not sure | 23 (3.87) |
| Counselling referral process (n=418) | I was self-referred | 209 (43.45) |
|  | I was referred by a HCP | 213 (44.28) |
|  | I am not sure how I was referred | 28 (5.82) |
|  | I have not had any counselling or therapy in the last 5 years | 31 (6.44) |
| Waiting time for counselling (n=481) | Less than a month | 104 (21.62) |
|  | Between 1 and 3 months | 133 (27.65) |
|  | Between 3 and 6 months | 96 (19.96) |
|  | More than 6 months | 105 (21.83) |
|  | I am not sure | 43 (8.94) |
| Effects of waiting time for counselling (>1 month) (n=334) | Symptoms got worse | 212 (63.47) |
|  | Seeking emergency mental health care | 79 (23.65) |
|  | Made day-to-day life harder | 95 (28.44) |
|  | Symptoms stayed the same | 93 (27.84) |
|  | Symptoms got better | 10 (2.99) |
|  | Other | 31 (9.28) |
| Ease of self-referral process (n=209) | Extremely easy | 55 (26.32) |
|  | Moderately easy | 74 (35.41) |
|  | Slightly easy | 30 (14.35) |
|  | Slightly difficult | 25 (11.96) |
|  | Moderately difficult | 14 (6.70) |
|  | Extremely difficult | 11 (5.26) |
| Reasons for difficulty of self-referral process (n=50) | I didn’t know where to look for help | 17 (34.00) |
|  | I didn’t know how to choose a counselling service | 22 (44.00) |
|  | I needed my GP to support my case but my GP did not support me | 11 (22.00) |
|  | Long waiting times | 37 (74.00) |
|  | Cost(s) | 12 (24.00) |
|  | Other | 16 (32.00) |
| Poor mental health affected work (n=290) | Yes | 256 (88.28) |
|  | No | 34 (11.72) |
| Effects of poor mental health on work (n=256) | Stopped working earlier than planned | 69 (26.95) |
|  | Impacted productivity | 145 (56.64) |
|  | Impacted work relationships | 145 (56.64) |
|  | Impacted earnings | 90 (35.16) |
|  | Impacted opportunities for promotion | 89 (34.77) |
|  | Increased stress levels | 226 (88.28) |
|  | Other | 63 (24.61) |
| Current workplace mental health services (n=263) | Training and support for mental health in the workplace | 86 (32.70) |
|  | Access to mental health apps (eg, Calm or Headspace) | 73 (27.76) |
|  | Private medical insurance | 30 (11.41) |
|  | Wellness programs | 89 (33.84) |
|  | Other | 30 (11.41) |
|  | I am not sure | 31 (11.79) |
|  | None | 81 (30.80) |
| Desired workplace mental health services (n=262) | Training and support for mental health in the workplace | 125 (47.53) |
|  | Access to mental health apps (eg, Calm or Headspace) | 110 (41.83) |
|  | Private medical insurance | 103 (39.16) |
|  | Wellness programs | 108 (41.06) |
|  | Other | 21 (7.98) |
|  | I am not sure | 44 (16.73) |
|  | None | 26 (9.89) |

***Note.*** GP, general practitioner; HCP, health care professional; NHS, national health service.
***Key.*** ^a^ Percentages add to more than 100% as respondents could select multiple options.

|  | |  |
| --- | --- | --- |
| **Table S2.** Experiences and interest in using digital technology for mental health (N=617) | | |
|  | | ***n* (%)** |
| Searched for help online for mental health symptoms | Yes | 545 (83.33) |
|  | No | 72 (11.67) |
| Types of help sought online (n=543)^a^ | Looked up mental health symptoms | 446 (82.14) |
|  | Looked up treatment or support options | 390 (71.82) |
|  | Took an online mental health test | 308 (56.72) |
|  | Looked up apps for mental health | 278 (51.20) |
|  | Looked for peer support groups | 254 (46.78) |
|  | Other | 40 (7.37) |
| Use of mental health apps (n=612) | Yes | 272 (44.44) |
|  | No | 340 (55.56) |
| Type of mental health apps (n=272) | Free | 206 (75.74) |
|  | Paid | 66 (24.26) |
| Desired mental health app features (n=596)^a^ | Help gain a better understanding of my mental health | 364 (61.07) |
|  | Self-help tips | 260 (43.62) |
|  | Monitor mental health symptoms | 314 (52.68) |
|  | To obtain a diagnosis | 184 (30.87) |
|  | Recommendations for treatment | 260 (43.62) |
|  | Referral to a HCP | 332 (55.70) |
|  | Other | 74 (12.42) |
| Prompt to use a mental health assessment app (n=594) | Referred by my HCP | 250 (42.09) |
|  | Gain access via workplace | 26 (4.38) |
|  | Search and use app independently | 318 (53.54) |
| Device preference (n=593) | Web | 389 (65.60) |
|  | Smartphone only | 204 (34.40) |
| Current device type (n=591) | Android | 299 (50.59) |
|  | iPhone | 246 (41.62) |
|  | Other | 15 (2.54) |
|  | I am not sure | 21 (3.55) |
|  | Prefer not to answer | 10 (1.69) |
| Browser type (n=590) | Google Chrome | 343 (58.14) |
|  | Mozilla Firefox | 28 (4.75) |
|  | Safari | 133 (22.54) |
|  | Microsoft Edge | 24 (4.07) |
|  | Opera | 5 (0.85) |
|  | Other | 18 (3.05) |
|  | I am not sure | 24 (4.07) |
|  | Prefer not to answer | 15 (2.54) |
| Interest in mental health assessment app and report preference (n=590) | Taking the report to your appointment with your healthcare provider to discuss | 186 (31.53) |
|  | The report being directly sent to your healthcare provider before your appointment | 289 (48.89) |
|  | No preference | 75 (12.71) |
|  | I would not be interested in taking the test in the first place | 40 (6.78) |
| Report preference (n=546)^a^ | Overall mental health severity score | 441 (80.77) |
|  | Indication of whether to seek further support | 454 (83.15) |
|  | Detailed list of mental health disorders that you have symptoms for | 376 (68.86) |
|  | Other | 54 (9.89) |
| Interest in psychiatrist reviewing the report (at a cost) (n=545) | Yes | 106 (19.45) |
|  | No | 256 (46.97) |
|  | I am not sure | 183 (33.58) |
| Would seek help if recommended by app (n=545) | Yes | 417 (76.51) |
|  | No | 21 (3.85) |
|  | I am not sure | 107 (19.63) |
| Who would you seek help from (n=523) | GP | 323 (61.76) |
|  | NHS psychological services (eg. IAPT) | 57 (10.90) |
|  | Psychiatrist | 40 (7.65) |
|  | Psychologist | 18 (3.44) |
|  | Therapist | 31 (5.93) |
|  | Mental health services provided via workplace | 14 (2.68) |
|  | Other | 29 (5.54) |
|  | None | 11 (2.10) |
| Comfortable with app using artificial intelligence to analyze mental health symptoms (n=542) | Extremely comfortable | 76 (14.02) |
|  | Comfortable | 181 (33.39) |
|  | Slightly comfortable | 120 (22.14) |
|  | Slightly uncomfortable | 85 (15.68) |
|  | Uncomfortable | 47 (8.67) |
|  | Extremely uncomfortable | 33 (6.09) |
| Willing to pay for mental health assessment app (n=575) | Yes | 160 (27.83) |
|  | No | 415 (72.17) |
| Smartwatch use (n=582) | Yes | 176 (30.24) |
|  | No | 406 (69.76) |
| Smartwatch purpose (n=176)^a^ | Track sleep | 106 (60.23) |
|  | Track physical activity levels | 149 (84.66) |
|  | Track heart rate | 111 (63.07) |
|  | Track mood | 15 (8.52) |
|  | Mindfulness or breathing exercises | 23 (13.07) |
|  | Workouts | 42 (23.86) |
|  | Track menstrual cycle | 24 (13.64) |
|  | Receive calls, texts, and app notifications | 70 (39.77) |
|  | Other | 11 (6.25) |

***Note.*** GP, general practitioner; HCP, health care professional; IAPT, Improving Access to Psychological Therapies; NHS, national health service.
***Key.*** ^a^ Percentages add to more than 100% as respondents could select multiple options.
